# The lived experience of HIV-infected patients in the face of a positive diagnosis of the disease: a phenomenological study

**DOI:** 10.1186/s12981-021-00421-4

**Published:** 2021-12-07

**Authors:** Behzad Imani, Shirdel Zandi, Salman khazaei, Mohamad Mirzaei

**Affiliations:** 1 Department of Operating Room, School of Paramedicine, Hamadan University of Medical Sciences, Hamadan, Iran; 2 Department of Operating Room, Student Research Committee, Hamadan University of Medical Sciences, Hamadan, Iran; 3 Research Center for Health Sciences, Hamadan University of Medical Sciences, Hamadan, Iran; 4 Department of Epidemiology, School of Public Health, Hamadan University of Medical Sciences, Hamadan, Iran

**Keywords:** AIDS, HIV, Lived experience, Phenomenological study

## Abstract

**Background:**

AIDS as a human crisis may lead to devastating psychological trauma and stress for patients. Therefore, it is necessary to study different aspects of their lives for better support and care. Accordingly, this study aimed to explain the lived experience of HIV-infected patients in the face of a positive diagnosis of the disease.

**Methods:**

This qualitative study is a descriptive phenomenological study. Sampling was done purposefully and participants were selected based on the inclusion and exclusion criteria. Data collection was conducted, using semi-structured interviews. Data analysis was performed using Colaizzi’s method.

**Results:**

12 AIDS patients participated in this study. As a result of data analysis, 5 main themes and 12 sub-themes were identified, which include**:** emotional shock (loathing, motivation of social isolation), the fear of the consequences (fear of the death, fear of loneliness, fear of disgrace), the feeling of the guilt (feeling of regret, feeling guilty, feeling of conscience-stricken), the discouragement (suicidal ideation, disappointment), and the escape from reality (denial, trying to hide).

**Conclusion:**

The results of this study showed that patients will experience unpleasant phenomenon in the face of the positive diagnosis of the disease and will be subjected to severe psychological pressures that require attention and support of medical and laboratory centers.

## Abstract (article highlights)

- Patients will experience severe psychological stress in the face of a positive diagnosis of HIV.
- Patients who are diagnosed with HIV are prone to make a blunder and dreadful decisions.
- AIDS patients need emotional and informational support when they receive a positive diagnosis.
- As a piece of bad news, presenting the positive diagnosis of HIV required the psychic preparation of the patient

## Introduction

HIV/AIDS pandemic is one of the most important economic, social, and human health problems in many countries of the world, whose, extent and dimensions are unfortunately ever-increasing [1]. In such circumstances, this phenomenon should be considered as a crisis, which seriously affects all aspects of the existence and life of patients and even the health of society [2]. Diagnosing and contracting HIV/AIDS puts a person in a vague and difficult situation. Patients suffer not only from the physical effects of the disease, but also from the disgraceful consequences of the disease. HIV/AIDS is usually associated with avoidable behaviors that are not socially acceptable, such as unhealthy sexual, relations and drug abuse: So the patients are usually held guilty for their illness [3]. On the other hand, the issue of disease stigma in the community is the cause of rejection and isolation of these patients, and in health care centers is a major obstacle to providing services to these patients [4]. Studies show that HIV/AIDS stigma has a completely negative effect on the quality of life of these patients [5]. Criminal attitudes towards these patients and disappointing behavior by family, community, and medical staff cause blame and discrimination in patients [6]. HIV/AIDS stigma is prevalent among diseases, making concealment a major problem in this behavioral disease. The stigma comes in two forms: a negative inner feeling and a negative feeling that other people in the community have towards the patient [7]. The findings of a study that conducted in Iran indicated that increasing HIV/AIDS-related stigma decreases quality of life of people living with HIV/AIDS [8]. Robert Beckman has defined bad news as “any news that seriously and unpleasantly affects persons’ attitudes toward their future”. He considers the impact of counseling on moderating a person’s feeling of being important [9]. Therefore, being infected by HIV / AIDS due to the stigma can be bad news, which will lead to unpleasant emotional reactions [10]. Studies that have examined the lives of these patients have shown that these patients will experience mental and living problems throughout their lives. These studies highlight the need for age-specific programming to increase HIV knowledge and coping, increase screening, and improve long-term planning [11, 12].

A prerequisite for any successful planning and intervention for people living with HIV/AIDS is approaching them and conducting in-depth interviews in order to discover their feelings, attitudes; their views on themselves, their illness, and others; and finally, their motivation to follow up and the participation in interventions [13]. Accordingly, the present study aimed to explain the lived experience of HIV-infected patients in the face of a positive diagnosis of the disease, since the better understanding of the phenomena leads to the smoother ways to help and care for these patients.

## Methods

### Study setting

In this study, a qualitative method of descriptive phenomenology was used to discover and interpret the lived experience of HIV-positive patients, when they face a positive diagnosis of the disease. The philosophical strengths underlying descriptive phenomenology afford a deeper understanding of the phenomenon being studied [14]. Husserl’s four steps of descriptive phenomenology were employed: bracketing, intuiting, analyzing and interpreting [15].

### Participants and sampling

Sampling was done purposefully and participants were selected based on inclusion criteria. In this purposeful sampling, participants were selected among those patients who had sufficient knowledge about this phenomenon. The sample size was not determined at the beginning of the study, instead, it continued until no new idea emerged and data-saturated. Participants were selected from patients who were admitted to the Shohala Behavioral Diseases Counseling Center in Hamadan-Iran. The center has been set up to conduct tests, consultations, medical and dental services, and to distribute medicines among the patients. Additional inclusion criteria for selecting a participant are: having a positive diagnosis experience at the center, Ability to recall events and mental thoughts in the face of the first positive diagnosis of the disease, having psychological and mental stability, having a favorable clinical condition, willingness to work with the research team, and the possibility of re-access for the second interview if needed. Exclusion criteria were unwillingness to participate in the study and inability of verbal communication in Persian language.

### Data collection

The interviews began with a non-structured question (tell us about your experience with a positive diagnosis) and continued with semi-structured questions. Each interview lasted 35–70 min and was conducted in two sessions if necessary. All interviews were conducted by the main investigator (ShZ) that who has experience in qualitative research and interviewing. The interview was recorded and then written down with permission of the participant.

### Data analysis

The descriptive Colaizzi method was used to analyses the collected data [16]. This method consists of seven steps: (1) collecting the participants’ descriptions, (2) understanding the meanings in depth, (3) extracting important sentences, (4) conceptualizing important themes, (5) categorizing the concepts and topics, (6) constructing comprehensive descriptions of the issues examined, and (7) validating the data following the four criteria set out by Lincoln and Guba.

### Rigor

Trustworthiness criteria were used to validate the research, due to the fact that importance of data and findings validity in qualitative research [17]. This study was based on four criteria of Lincoln and Guba: credibility, transferability, dependability, and conformability [18]. For data credibility, prolong engagement and follow-up observations, as well as samplings with maximum variability were used. For dependability of the data, the researchers were divided into two groups and the research was conducted as two separate studies. At the same time, another researcher with the most familiarity and ability in conducting qualitative research, supervised the study as an external observer. Concerning the conformability, the researchers tried not to influence their own opinions in the coding process. Moreover, the codes were readout by the participants as well as two researcher colleagues with the help of an independent researcher and expert familiar with qualitative research. Transferability of data was confirmed by offering a comprehensive description of the subject, participants, data collection, and data analysis.

### Ethical considerations (ethical approval)

The present study was registered with the ethics code IR.UMSHA.REC.1398.1000 in Hamadan University of Medical Sciences. The purpose of the study was explained and all participants’ consents were obtained at first step. All participants were assured that the information obtained would remain confidential and no personal information would be disclosed. Participants were also told that there was no need to provide any personal information to the interviewer, including name, surname, phone number and address. To gain more trust, interviews were conducted by a person who was not resident of Hamadan and was not a native of the region, this case was also reported to the participants.

## Results

Twelve HIV-infected participated in this study. The mean age of the participants was 36.41 ± 4.12 years. 58.33% of the participants were male and 41.66% were married. Of these, 2 were illiterate, 2 had elementary diploma, 6 had high school diploma and 2 had academic education. Six of them were unemployed, 5 were self-employed and 1 was an official employee. These people had been infected by this disease for 6.08 ± 2.71 years, in average (Table 1).

**Table 1:** The demographic characteristics of participants

Participants	Gender	Age	Marital status	Education	Job	Duration of infection
P1	Male	38	Single	Diploma	Self-employed	7
P2	Female	41	Married	Elementary	Unemployed	6
P3	Female	36	Married	Diploma	Unemployed	5
P4	Male	37	Single	Academic	Employee	6
P5	Female	42	Single	Illiterate	Unemployed	11
P6	Male	38	Married	Diploma	Self-employed	8
P7	Male	32	Single	Academic	Self-employed	4
P8	Male	39	Married	Diploma	Self-employed	7
P9	Female	29	Single	Diploma	Unemployed	2
P10	Female	40	Single	Elementary	Unemployed	10
P11	Male	31	Single	Diploma	Self-employed	4
P12	Female	34	Married	Illiterate	Unemployed	3

Analysis of the HIV-infected patients’ experiences of facing the positive diagnosis of the disease by descriptive phenomenology revealed five main themes: emotional shock, the fear of the consequences, the feeling of the guilt, the discouragement, and the escape from reality (Table 2).

**Table 2:** The themes and sub-thems

Themes	Sub-themes
1. Emotional shock	Loathing
	Motivation of social isolation
2. The fear of the consequences	Fear of the death
	Fear of loneliness
	Fear of disgrace
3. The feeling of the guilt	Feeling of regret
	Feeling guilty
	Feeling of conscience-stricken
4. The discouragement	Suicidal ideation
	Disappointment
5. The escape from reality	Denial
	Trying to hide

### Emotional shock

Emotional shock is one of the unpleasant events that these patients have experienced after facing a positive diagnosis of the disease. This experience has manifested in loathing and motivation of social isolation.

#### Loathing

These patients stated that after facing a positive diagnosis of the disease, they developed a strong inner feeling of hatred towards the source of infection. The patients feel hatred, since they hold the carrier as responsible for their infection. *“…After realizing I was affected, I felt very upset with my husband, I did not want to see him again, because it made me miserable, I even decided to divorce ….”(P3).*

#### Motivation of social isolation

The experiences of these patients showed that after facing the incident, they have suffered an internal failure that has caused them to try to distance from other people. These patients have become isolated, withdrawing from the community and sometimes even from their families. *“…After this incident, I decided to live alone forever and stay away from all my family members. I made a good excuse and broke up our engagement…” (P7).*

### Fear of the consequences

Fear of the consequences is one of the unpleasant experiences that these patients will face, as soon as they receive a positive diagnosis of the disease. Based on experiences, these patients feel fear of loneliness, death, and disgrace as soon as they hear the positive diagnosis.

#### Fear of the death

The patients said that as soon as they got the positive test results, they thought that the disease was incurable and would end their lives soon. *“…When I found I had AIDS, I was very upset and moved like a dead man because I was really afraid that at any moment this disease might kill me and I would die …” (P1).*

#### Fear of loneliness

The participants stated that one of the feelings that they experienced as soon as they received a positive diagnosis of the disease was the fear of being alone. They stated that at that moment, the thought of being excluded from society and losing their intimacy with them was very disturbing. *“…The thought that I could no longer have a family and had to stay single forever bothered me a lot, it was terrifying to me when I thought that society could no longer accept me as a normal person …” (P10).*

#### Fear of disgrace

One of the feelings that these patients experienced when faced the positive diagnosis of the disease was the fear of disgrace. They suffer from the perception that the spread of news of the illness hurts the attitudes of those around them and causes them to be discredited. *“…It was very annoying for me when I thought I would no longer be seen as a member of my family, I felt I would no longer have a reputation and everyone would think badly of me …” (P2).*

### Feeling of the guilt

From other experiences of these patients in facing the positive diagnosis of the disease is feeling guilty. This feeling appears in patients as feeling of regret, guilty and remorse.

#### Feeling of regret

These patients stated that they felt remorse for their lifestyle and actions as soon as they heard the positive diagnosis of the disease, because they thought that if they had lived healthier, they would not have been infected. *“…After realizing this disease, I was very sorry for my past, because I really did not have a healthy life. I made a series of mistakes that caused me to get caught. At that moment, I just regretted why I had this disaster …” (P11).*

#### Feeling guilty

The experience of these patients has shown that after receiving a positive diagnosis of the disease, they consider themselves guilty and complain about themselves. These patients condemn their lifestyle and sometimes even consider themselves deserving of the disease and think that it is a ransom that they have paid back. *“…after getting the disease, I realized that I was paying the ransom because I was hundred percent guilty, I was the one who caused this situation with a series of bad deeds, and now I have to be punished …” (P5).*

#### Feeling of conscience-stricken

One of the experiences that these patients reported is the pangs of conscience. These patients stated that after receiving a positive diagnosis of the disease, the thought that as a carrier they might have contaminated those around them was very unpleasant and greatly affected their psyche. *“…after getting the disease. It was shocked and I was just crazy about the fact that if my wife and children had taken this disease from me, what would I do, I made them hapless … and this as very annoying for me …” (P8).*

### Discouragement

Discouragement is an unpleasant experience that patients experienced after receiving a positive HIV test results. Discouragement in these patients appears in the suicidal ideation and disappointment.

#### Suicidal ideation

The patients stated that they were so upset with the positive diagnosis of the illness and they immediately thought they could not live with the fact and the best thing to do was to end their own lives. *“…The news was so bad for me that I immediately thought that if the test result was correct and I had AIDS, I would have to kill myself and end this wretch life, oh, I had a lot of problem and the thought of having to wait for a gradual death was horrible to me …” (P12).*

#### Disappointment

The experience of these patients shows that a positive diagnosis of the disease for these patients leads to a destructive feeling of disappointment. So that they are completely discouraged from their lives. These patients think that their dreams and goals are vanished and that they have reached the end and everything is over. *“…It was a horrible experience, so at that moment I felt my life was over, I had to prepare myself for a gradual death, I was at marriage ages when I thought I could no longer get married, I saw life as meaningless …” (P7).*

### Escape from reality

The lived experience of these patients shows that after receiving a positive diagnosis of the disease, they found that this fact was difficult to accept and somehow tried to escape from the reality. This experience has been in the form of denial and trying to hide from others.

#### Denial

One of the experiences of these patients in dealing with the positive test result of this disease has been to deny it. In this way, patients believed that the test result was wrong or that the result belonged to someone else. For this reason, the patients referred to other laboratories after receiving the first positive diagnosis of the disease. *“…After the lab told me this and found out what the disease really was, I was really shocked and said it was impossible, it was definitely wrong and it is not true … I could not believe it at all, because I was a professional athlete and this could not happen to me. So I immediately went to a bigger city and there I went to a few laboratories for further tests …” (P6).*

#### Trying to hide

These patients stated that after receiving the first positive diagnosis of the disease, they thought that no one should notice their disease and should remain anonymous as much as possible. *“…I immediately decided that no one in my city should know that I got this disease and the news should not be spread anywhere, so I discard my phone number through which our city laboratory communicated with me and I came here to do a re-examination and go to the doctor, and after all these years, I always come here again for an examination …” (P4).*

## Discussion

In this qualitative study, we attempted to discover lived experience of HIV-infected patients in the face of a positive diagnosis of the disease. Therefore, a descriptive phenomenological method was applied. As a result of this study, based on the experiences of the HIV-infected patients, the five main themes of emotional shock, fear of the consequences, feelings of guilt, discouragement and, escape from reality were obtained.

In this study, it was shown that the confrontation of these patients with the positive diagnosis of the disease causes them to experience a severe emotional shock. In this regard, Yangyang Qiu et al. [19] argued that anxiety and depression are very common among HIV-infected patients who have recently been diagnosed with the disease. The experience of the participants has shown that this emotional shock appears in the form of loathing and the motivation of social isolation. In fact, in these patients, the feeling of the loathing is an emotional response to the primary carrier that has infected them. The study of Imani et al. [20] have shown that decrease emotional intelligence in an environment where there is an HIV carrier, other people hate him/her, because they see him/her as a risk factor for their infection. The experience of the participants has also shown that receiving a positive diagnosis will motivate social isolation in these patients. Various studies have revealed that one of the consequences of AIDS/HIV that patients will suffer from, is social isolation [21, 22].

Another experience of the participants, according to this study is fear of the consequences. This phenomenon appears in these patients as fear of the death, fear of loneliness, and fear of disgrace. Due to the nature of the disease, these patients feel an inner fear of premature death, as soon as they receive a positive diagnosis. In this regard, the study of Audrey K Miller et al. [23] showed that death anxiety in AIDS patients is a psychological complication. the participants have stated that they are very afraid of being alone after receiving a positive diagnosis, which is a natural feeling according to Keith Cherry and David H. Smith [24]; because these patients will mainly experience some degree of loneliness. HIV-infected patients also experienced a fear of disgrace, which will go back to the nature of the disease and people’s insight; but they should be aware that, as Newman Amy states, AIDS/ HIV is a disease, not a scandal [25].

Another experience of the participants in dealing with the positive diagnosis of the disease is guilt feeling. The patients will experience feelings of regret, the feeling guilty and feeling of the conscience-stricken. The experience of the participants shows that they regret their past. Earlier studies have also revealed that regret for the past is a common phenomenon among the patients living with HIV [26–28]. HIV-infected feel guilty while facing the positive diagnosis of the disease and consider themselves the main culprit of the situation. They often play a direct role in their infection, and their past lifestyle for sure [29]. Our study also found that these patients feel the conscience-stricken after a positive diagnosis, because they suspect that they may have infected people around them. This disease can be easily transmitted from the carrier to others if the health protocols are not followed [30–32].

Another experience of HIV-infected in dealing with the receiving a positive diagnosis of the disease is discouragement. These patients are disappointed and sometimes decide to suicide. Based on the lived experience of HIV-infected, it was found that receiving a positive diagnosis of the disease, will discourage them from life and patients will be disappointed in many aspects of life. Studies have shown that AIDS/HIV, as a crisis, will greatly reduce the patients' life expectancy and that they will continue to live in despair [33]. Studies also stated that they considered suicide as a solution to relieve stress when receiving a positive diagnosis. In this regard, various studies have emphasized that among the AIDS/HIV patients, loss of self-esteem and severe stress have led to high suicide rates [34–36].

According to the patients, trying to escape from reality is another phenomenon that they will experience. This phenomenon will occur in patients as denial and trying to hide the disease from others. Based on the lived experience of these patients, it was found that after facing a positive diagnosis, HIV-infected tend to deny that they are infected. In this regard, various studies have shown that AIDS/HIV patients in different stages of the disease and their lives try to deny it in different ways [37–39]. The HIV-infected also stated that at the beginning of the positive diagnosis of the disease, did not want others to know, so they wanted to hide themselves from others in any way possible. In this regard, Emilie Henry et al. [40] have shown that a high percentage of the patients living with AIDS/HIV have tried that others do not notice that they are ill.

One of the strengths of this study is the methodology of the study, because in this study, an attempt has been made to use descriptive phenomenology to explain the lived experience of HIV-infected patients when faced with a positive diagnosis of this disease. In fact, in this study, patients' experience of this particular situation was identified, and with careful analysis, the experiences of these people became codes and concepts, each of which can be a bridge that keeps the path of modern knowledge open to help these patients. One of the limitations of this study is the generalizability of the findings because patients’ experiences in different societies that have cultural, religious, subsistence, and economic differences can be different.

## Conclusion

The results of this study showed that patients will experience unpleasant experiences in the face of receiving a positive diagnosis of the HIV. Patients’ unpleasant experiences at that moment include emotional shock, fear of the consequences, feeling guilty, discouragement and escape from reality. Therefore, medical and laboratory centers must pay attention to the patients' lived experience, and try to support the patients through education, counseling and other support programs to minimize the psychological trauma caused by the disease.

## Data Availability

The datasets used and analyzed during the current study are available from the corresponding authors through reasonable request.

## Abbreviations

AIDS: Acquired immunodeficiency syndrome
HIV: Human immunodeficiency virus
